# Effects of electroacupuncture on imaging and behavior in rats with ischemic stroke through miR-212-5p

**DOI:** 10.1186/s12868-023-00827-y

**Published:** 2023-12-06

**Authors:** Sisi Li, Xiangxin Xing, Xuyun Hua, Yuwen Zhang, Jiajia Wu, Chunlei Shan, Mouxiong Zheng, He Wang, Jianguang Xu

**Affiliations:** 1 School of Rehabilitation Science, Shanghai University of Traditional Chinese Medicine, NO. 1200, Cailun Road, Shanghai, 201203 Shanghai China; 2 Department of Physical Medicine and Rehabilitation, The Second Affiliated Hospital, Yuying Children’s Hospital of Wenzhou Medical University, Wenzhou, 325027 China; 3 Center of Rehabilitation Medicine, Yueyang Hospital of Integrated Traditional Chinese and Western Medicine, Shanghai University of Traditional Chinese Medicine, Shanghai, 200437 China; 4 Department of Traumatology and Orthopedics, Yueyang Hospital of Integrated Traditional Chinese and Western Medicine, Shanghai University of Traditional Chinese Medicine, Shanghai, 200437 China; 5 Institute of Science and Technology for Brain-Inspired Intelligence, Fudan University, Shanghai, 201203 China; 6 Engineering Research Center of Traditional Chinese Medicine Intelligent Rehabilitation, Ministry of Education, Shanghai, 201203 China

**Keywords:** Electroacupuncture, Ischemic Stroke, Amplitude of low frequency fluctuations, Regional homogeneity, MiR-212-5p

## Abstract

**Background:**

Ischemic stroke is a serious disease leading to significant disability in humans worldwide. Increasing evidence suggests that some microRNAs (miRNAs) participate in the pathophysiology of ischemic stroke. A key role for MiR-212 has been found in neuronal function and synaptic plasticity. Ischemic stroke can be effectively treated with electroacupuncture (EA); however, there is a lack of understanding of the relevant mechanisms. In this study, we employed behavioral test and resting-state functional magnetic resonance imaging (rs-fMRI) to detect behavioral and brain function alterations in rats suffering from ischemic stroke. The efficacy of EA therapy and miR-212-5p’s role in this process were also evaluated.

**Methods and results:**

Forty rats were randomly divided into the following groups: Sham, middle cerebral artery occlusion/reperfusion (MCAO/R), MCAO/R + EA, MCAO/R + EA + antagomir-negative control and MCAO/R + EA + antagomir-212-5p groups. Behavioral changes were assessed by Catwalk gait analysis prior to and after modeling. Rs-fMRI was performed at one week after EA treatment, amplitude of low-frequency fluctuations (ALFF) and regional homogeneity (ReHo) were calculated to reveal neural activity. Furthermore, neuronal apoptosis in the ischemic penumbra was analyzed using a TUNEL assay. Treatment with EA significantly improved the performance of rats in the behavioral test. The motor and cognition-related brain regions showed decreased ALFF and ReHo following focal cerebral ischemia-reperfusion, and EA treatment could reactivate these brain regions. Moreover, EA treatment significantly decreased MCAO/R-induced cell death. However, the transfection of antagomir-212-5p attenuated the therapeutic effect of EA.

**Conclusions:**

In conclusion, the results suggested that EA improved the behavioral and imaging outcomes of ischemic stroke through miR-212-5p.

## Background

Stroke is a major cause of neurological injury and death [1]. Ischemic stroke, which represents approximately 80% of all strokes, usually causes severe neuronal damage and loss of neuronal function [2]. At present, an important goal of neurorehabilitation is promoting the recovery of neuronal plasticity and motor function of the affected limb. MicroRNAs (miRNAs) are a large class of short (approximately 22 nt) noncoding RNAs found in the body [3]. The miRNA family is the most diverse group of gene expression transcriptional regulators involved in the regulation of a variety of biological processes, including apoptosis, cell proliferation, cellular invasion, angiogenesis, tumor proliferation, metastasis and neural plasticity [4, 5]. Many miRNAs have been reported that act as potential biomarkers and play an important role in the pathogenesis of ischemic stroke [6]. MiR-212-5p is a widely distributed miRNA in the brain and has been recently found to contribute to the the development of the nervous system and synaptic plasticity [7].

Electroacupuncture (EA) is an important therapeutic means in traditional Chinese medicine and has been extensively used worldwide for the treatment of numerous diseases. It has been considered a potentially effective strategy to facilitate motor function recovery after stroke [8]. Clinically, Quchi (LI11) and Zusanli (ST36) are frequently used for the stroke treatment [9]. With the widespread use of EA in clinical practice, many studies have been performed to examine the possible mechanisms of EA for functional recovery poststroke. Resting-state functional magnetic resonance imaging (rs-fMRI), relies on signals from blood oxygen levels, is commonly used to evaluate spontaneous neuronal activity in the brain during rest [10]. Amplitude of the low-frequency fluctuations (ALFF) and regional homogeneity (ReHo) are often used to reflect spontaneous changes in neural activity and brain function [11, 12]. ReHo measures the synchronization or similarity of the time courses with its neighboring voxels, while ALFF depicts the magnitude of the fluctuation of every signal time course [13].

In this study, we employed an in vivo model of middle cerebral artery occlusion/reperfusion (MCAO/R). To assess the effectiveness of EA therapy on motor function recovery, we conducted the Catwalk gait analysis, and applied ALFF and ReHo to reveal abnormal local activity in brain regions. We aimed to test the hypothesis that whether miR-212-5p contributes to the therapeutic effects of EA on MCAO/R and to explore the influence of EA on spontaneous regional brain activity in rats suffering from ischemic stroke.

## Methods

### Animals

Healthy male Sprague–Dawley rats (260–280 g) were obtained from Shanghai Laboratory Animal Research Center (Shanghai, China). We used only male rats in this study because previous research demonstrated that oestrogens protect against cerebral ischemia in animal models [14]. The rats were housed under controlled environmental conditions (12:12 h light/dark cycle, 40–50% humidity, 23 ± 2 °C ambient temperature) with standard chow and water. The experimental protocol was reviewed and approved by the Committee on Animal Care and Usage of Shanghai University of Traditional Chinese Medicine (approval No. PZSHUTCM200110002). The present study was performed following the National Institutes of Health Guide for the Care and Use of Laboratory Animals. All sections of this report adhere to the ARRIVE Guidelines for reporting animal research. A great deal of effort was made to minimize suffering for animals. According to the random digital table method, forty rats were randomly divided into five groups (n = 8 each group): Sham group, MCAO/R group, MCAO/R + EA group, MCAO/R + EA + antagomir-negative control (NC) group and MCAO/R + EA + antagomir-212-5p group. The number of rats for each groups was calculated based on previous literature [15]. When the rat appeared extreme emaciation [16, 17], the rat underwent euthanasia as the humanitarian end point of the study. No animals were excluded from this study. Upon completion of the study, the rats were anesthetized (pentobarbital sodium, 30 mg/kg) by intraperitoneal injection and euthanized.

### Intracerebroventricular injection

The antagomir-212-5p and antagomir-NC were designed and synthesized by GenePharma (Shanghai, China) and were diluted with DEPC according to the instructions. Subsequently, the antagomir-212-5p or antagomir-NC (10 µM in 7 µl) [18] was delivered by intracerebroventricular injection (ICV). Coordinates for ICV were relative to the location of the bregma: posterior 1 mm, lateral 1.5 mm (left side), depth 3.5 mm. Antagomir-212-5p and antagomir-NC were injected slowly over a period of 5 min, after which a further 5 min were spent holding the needle in place, followed by slow needle withdrawal. Subsequently, the scalp was closed with sutures.

### Focal cerebral ischemia reperfusion model

We established the MCAO/R model by briefly blocking the left side for 2 h and then performing refusion according to the method of Longa [19]. Rats were weighed and anesthetized with 3% pentobarbital sodium at 30 mg/kg pentobarbital intraperitoneally. The ipsilateral common carotid artery, external carotid artery (ECA) and internal carotid artery (ICA) were separated from connective tissues. After ligation of the distal end of the ECA, a small incision was performed, and a monofilament nylon suture (Guangzhou Jia Ling Biotechnology Co., Ltd., Guangzhou, China) was interleaved via the ECA into the ICA until slight resistance was felt. After 2 h of occlusion, the monofilament nylon was gently withdrawn to restore blood flow supply to the MCA area. The Sham groups underwent the same procedure but without the monofilament nylon suture insertion.

### Acupuncture intervention

Acupuncture intervention was carried out at approximately 9:00 A.M. each day. An immobilization apparatus was used to fix the rat’s body, while allowing them to move their heads and limbs freely. In order to reduce anxiety, rats were acclimated to the immobilization apparatus at least 3 days before acupuncture intervention. We inserted sterile disposable stainless-steel needles of 0.25 × 13 mm into LI11 and ST36 on the contralateral side of the brain. For 7 consecutive days, EA was conducted for 30 min once a day, with a frequency of 2/15 Hz, using an EA apparatus (HANS-200, Nanjing Jisheng Medical Co., Ltd., Nanjing, China).

### Catwalk gait analysis

The motor performance and coordination of animals were assessed using an automated quantitative gait analysis system (Catwalk™, Wageningen, Netherlands). Each group included eight rats. Behavioral tests were conducted by researchers who were blind to grouping. A quiet, darkened room was used for the experiment. Prior to experimentation, each rat performed three trials without any interruption to cross the runway of the Catwalk system. An 150-cm-long runway was constructed with a glass platform covered by a black tunnel and a food reward at one end for the rats. This test was performed before surgery and on days 1, 3, 5, and 7 following the surgery, respectively. Data were acquired and analysed using Catwalk version 10.6 software.

### Motor-evoked potential (MEP)

Electrophysiological tests were performed for each group by using an electromyography-evoked potentiometer (9033A07, Keypoint; Medronic, Skovlunde, Denmark) at 7 days following the surgery. Each group included six rats. Rats were anesthetized for electrophysiological testing, single electrical pulses of 100 µs were applied, and MEP was recorded with an electrode from the right biceps brachii muscle. The stimulation intensity was increased gradually until no further increase in the amplitude was observed.

### FMRI data acquisition

All animal studies were performed using a 11.7 T animal scanner (Bruker Corporation, Germany). FMRI scans were obtained from the rats at 7 days after EA treatment. Each group included eight rats. The animals were deeply anesthetized with isoflurane (5% isoflurane for anesthesia induction; 1.5% isoflurane combined with 0.05 mg/kg dexmedetomidine for maintenance), placed in a prone position and fixed on the scanner. The rs-fMRI scans were acquired using an echo-planar imaging sequence: flip angle = 90°, slice thickness = 0.3 mm, number of averages = 1, repetition time = 3000 ms, echo time = 8.142 ms, and field of vision = 27 × 27 mm^2^. The quality of the scanned images was checked immediately after each scan, and rescans were performed if the images did not meet the requirements.

### FMRI data processing

The preprocessing of images and subsequent analyses were performed using the statistical parametric mapping (SPM) 8 toolbox (http://www.fil.ion.ucl.ac.uk/spm/) on the MATLAB 2013b platform. All images were converted to NIfTI format. For fMRI data preprocessing, we removed the first 10 time points due to the possible instability of the initial MRI signal. Slice timing correction, coregistration, and realignment for head motion correction were followed. Nonbrain tissue was removed using the Micron tool and manual reorientation to the anterior-posterior commissure plane. The fMRI data were then normalized to the standard template and resampled to a 2.06 × 2.06 × 2 mm voxel size. Finally, we smoothed the images by a full width at half maximum triploid as the voxel size (6.18 × 6.18 × 6 mm), which improved the signal-to-noise ratio. Subsequently, in addition to analyze the ALFF, temporally bandpass filtering and linear detrending of the time series were performed for each voxel to reduce low-frequency drift and physiological high-frequency respiration. We regressed out nuisance variables, including the Friston 24 motion parameters [20], cerebrospinal fluid signal and white matter signal.

### ALFF and ReHo analysis

ALFF and ReHo calculations were performed using REST software (Beijing Normal University, http://www.restfmri.net). For ALFF calculations, in order to obtain the power spectrum for each voxel, the time series of each voxel was converted to frequency domain through a fast Fourier transform. Based on the fact that power of a specified frequency is relative to the square of the amplitude of this frequency, after obtaining the power spectrum, we calculated the averaged square root (i.e., ALFF value) at each frequency of the power spectrum [21]. We converted each individual’s ALFF value to Z scores to allow between-group comparisons.

Individual ReHo maps were generated by calculating Kendall’s coefficient of concordance of the time series of each voxel and its 26 neighboring voxels [12].

To normalize the ReHo map further, we divided each voxel’s ReHo value by the global mean ReHo value. Last, the ReHo data was smoothed with a Gaussian kernel with a full width at half maximum of 6.18 × 6.18 × 6 mm. Using two-sample t tests, the ALFF/ReHo data were compared between groups.

### TUNEL analysis

After the experimental period, rats were transcardially perfused with 0.9% NaCl, followed by 4% paraformaldehyde (PFA), after which the brains were removed and postfixed overnight in 4% PFA. Each group included 3 rats. The brain tissues were dehydrated, embedded in paraffin, and cut into 5-µm-thick coronal sections. As per the manufacturer’s instructions, we assessed the number of apoptotic neurons following MCAO/R injury using terminal transferase-mediated dUTP nick end-labeling (TUNEL) staining. Neurons in the ischemic penumbra region were observed under a light microscope, and the apoptotic nuclei were stained brown.

### Statistical analysis

Statistical analysis was performed with SPSS software (SPSS Standard version 22.0, SPSS), and the results are expressed as the mean ± standard error of the mean (SEM). The data were analyzed by one-way analysis of variance followed by least significant difference test (equal variances assumed) or Dunnett’s T3 test (equal variances not assumed). The significance level was set at *P* < 0.05.

## Results

### EA treatment significantly promoted gait functional recovery via miR-212-5p

**Fig. 1:**
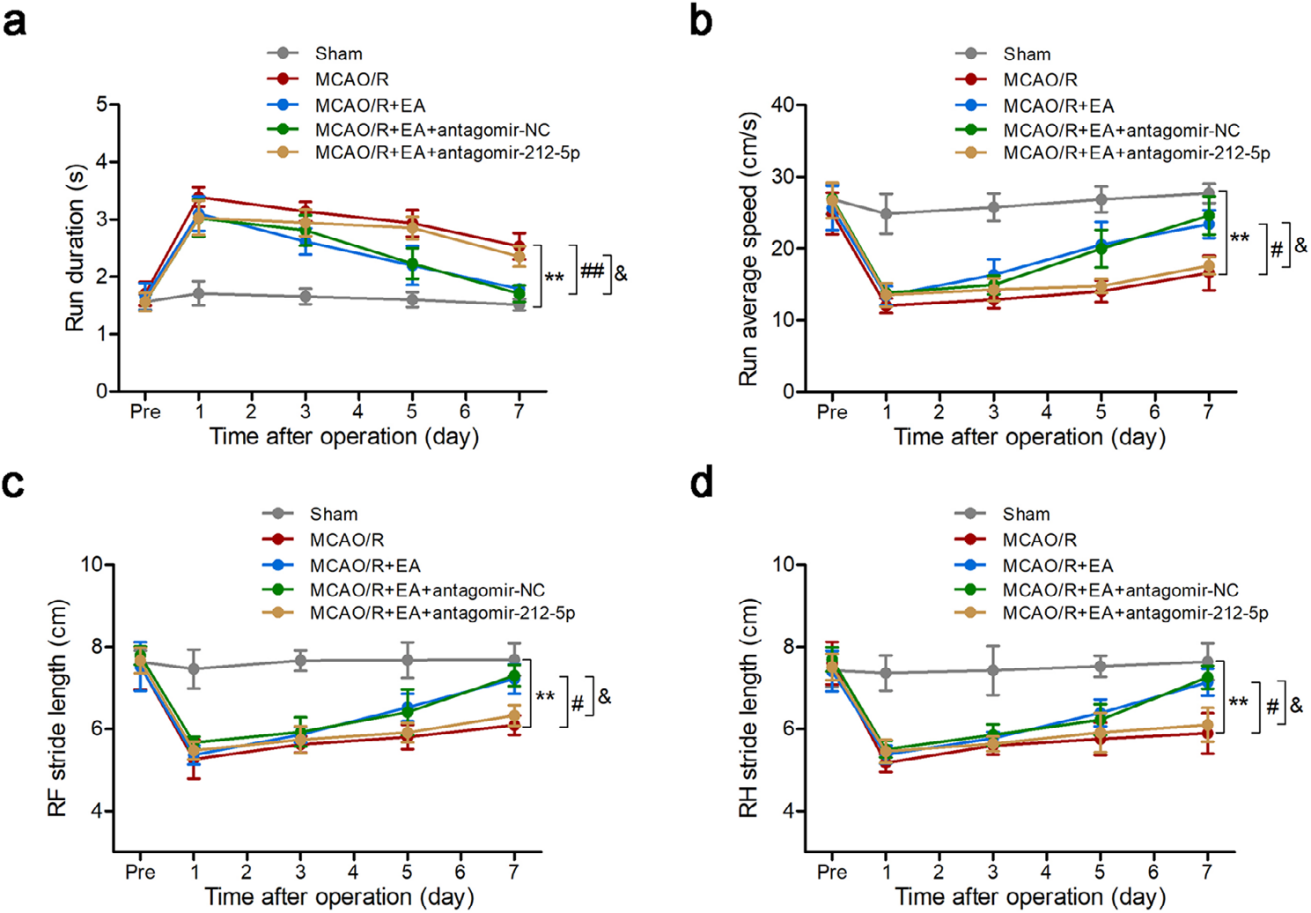
Treatment with EA promoted gait functional recovery via miR-212-5p after MCAO/R. At 7 days after modeling, rats showed slower average running speed and reduced stride length in the affected limbs compared with the Sham group. The rats treated with EA reduced the running duration and improved stride length compared with the MCAO/R group. These beneficial effects of EA were attenuated by treatment with antagomir-212-5p. **a** Run duration. **b** Run average speed. **c** RF stride length. **d** RH stride length. The data are presented as the means ± SEM (n = 8 per group). ^*^P < 0.05 and ^**^P < 0.01 compared with the Sham group, ^#^*P* < 0.05 and ^##^*P* < 0.01 compared with the MCAO/R group, ^&^*P* < 0.05 and ^&&^*P* < 0.01 compared with the MCAO/R + EA group

Catwalk gait analysis was conducted to evaluate the effect of EA on motor function (Fig. 1). MCAO/R group showed slower average running speed and longer durations of running on day 7 compared with the Sham group (*P* < 0.001). Meanwhile, MCAO/R group exhibited severely reduced stride length in the affected limbs (*P* < 0.001). The rats treated with EA reduced the running duration and improved stride length compared with the rats subjected to MCAO/R (*P* < 0.05). There were no significant differences between the MCAO/R + EA and MCAO/R + EA + antagomir-NC groups. However, these beneficial effects of EA were attenuated by treatment with antagomir-212-5p, suggesting that the effect of EA treatment might have partly operated through miR-212-5p.

### EA ameliorates abnormal motor-evoked potential through miR-212-5p

**Fig. 2:**
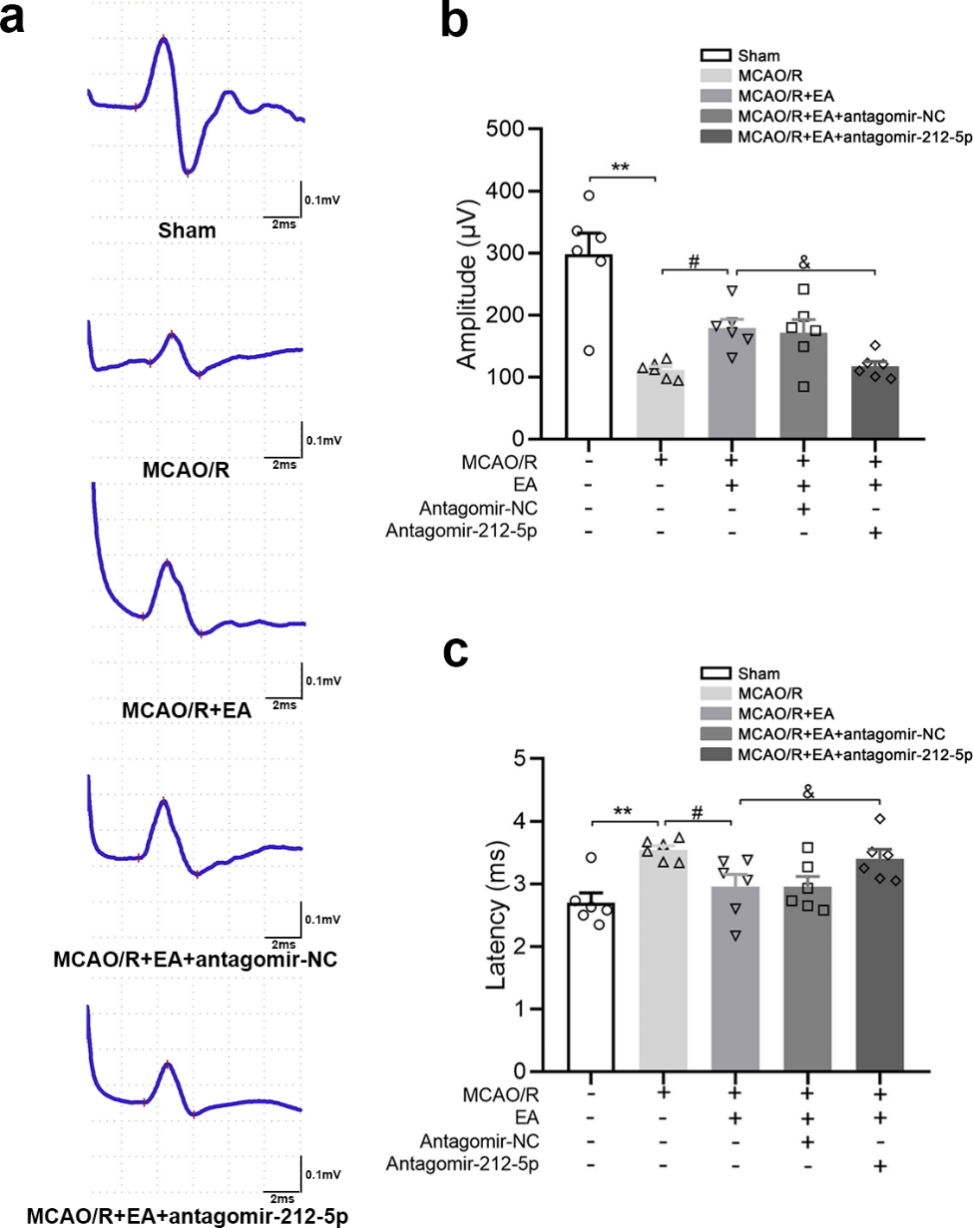
EA ameliorates abnormal motor evoked potential through miR-212-5p at 7 days after MCAO/R. **a** Illustrative waveforms of motor evoked potential (MEP) for each group. **b** The amplitude of MEP for each group. **c** The latency of MEP for each group. At 7 days after modeling, animals showed lengthened MEP onset latency and reduced peak amplitude post-surgery. The peak amplitude of MEP in the MCAO/R + EA group was significantly increased compared with the MCAO/R group, while, the onset latency of MCAO/R + EA group was significantly shorter than the MCAO/R group. No significant differences between MCAO/R + EA group and MCAO/R + EA + antagomir-NC group were found. Antagomir-212-5p attenuated EA effect on latency and peak amplitude. +: with indicated treatment; −: without indicated treatment. The data are presented as the means ± SEM (n = 6 per group). ^*^P < 0.05 and ^**^P < 0.01 compared with the Sham group, ^#^*P* < 0.05 and ^##^*P* < 0.01 compared with the MCAO/R group, ^&^*P* < 0.05 and ^&&^*P* < 0.01 compared with the MCAO/R + EA group

At 7 days after modeling, nerve conduction in all rats was assessed by MEP, which was analyzed by the onset latency and peak amplitude. As shown in Fig. 2, animals may show a lengthened MEP onset latency and reduced peak amplitude postsurgery. The peak amplitude of MEP was significantly increased in the MCAO/R + EA group compared with the MCAO/R group (*P* < 0.05), while the onset latency was significantly shorter in the MCAO/R + EA group than in the MCAO/R group (*P* < 0.05). No significant differences were found between the MCAO/R + EA and MCAO/R + EA + antagomir-NC groups. Antagomir-212-5p attenuated the EA effect on the latency and peak amplitude (*P* < 0.05). Therefore, the improvements in electrophysiology demonstrated that EA ameliorated the abnormal latency and peak amplitude through miR-212-5p.

### Comparison of ALFF and ReHo reveals changes in brain function

**Fig. 3:**
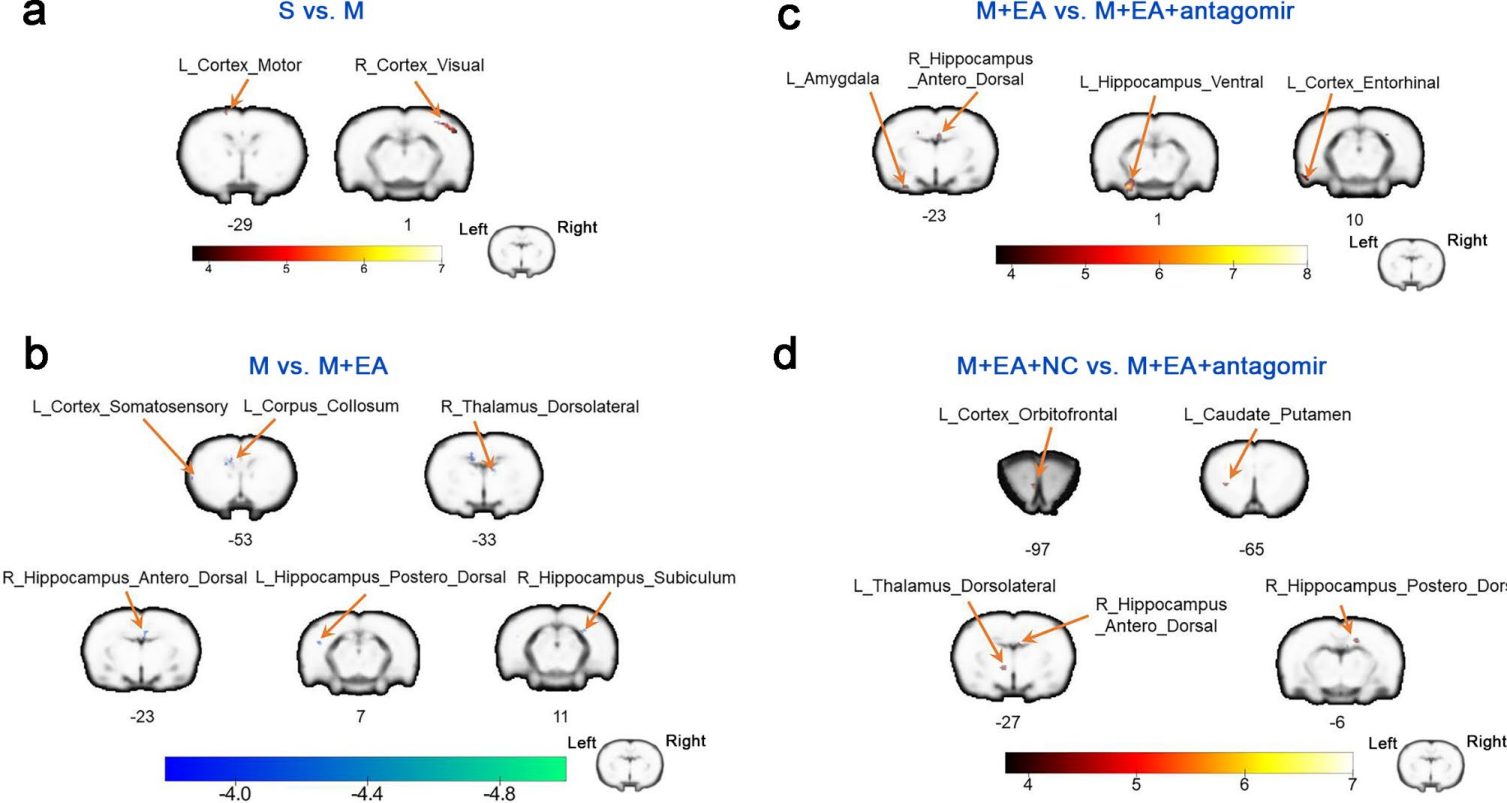
Difference in the regional homogeneity (ReHo) among groups. **a** Altered ReHo in the brain regions of MCAO/R group compared to the Sham group. **b** Altered ReHo in the brain regions of MCAO/R + EA group compared to the MCAO/R group. **c** Altered ReHo in the brain regions of MCAO/R + EA + antagomir-212-5p group compared to the MCAO/R + EA group. **d** Altered ReHo in the brain regions of MCAO/R + EA + antagomir-212-5p group compared to the MCAO/R + EA + antagomir-NC group. n = 8 per group. The voxel-level height threshold was *P* < 0.001(uncorrected) and the cluster-extent threshold were 10 voxels. S, Sham group; M, MCAO/R group; M + EA, MCAO/R + EA group; M + EA + NC, MCAO/R + EA + antagomir-NC group; M + EA + antagomir, MCAO/R + EA + antagomir-212-5p group; L, left; R, right

**Fig. 4:**
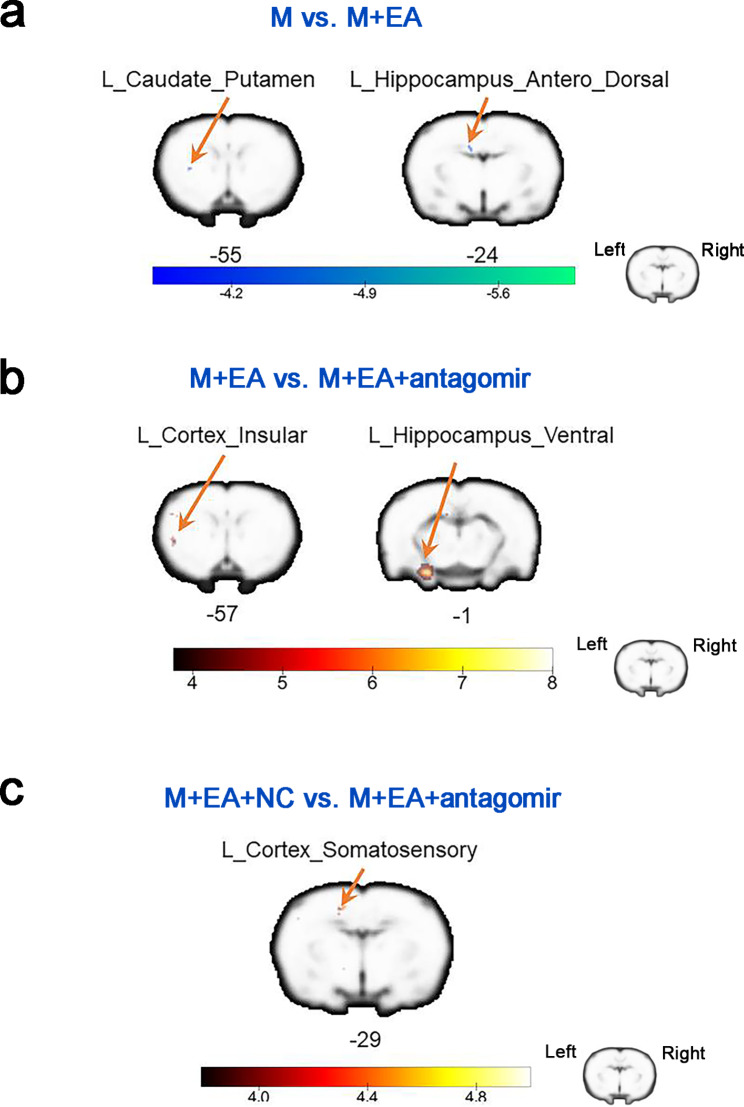
Difference in the amplitude of low-frequency fluctuations (ALFF) among groups. **a** Altered ALFF in the brain regions of MCAO/R + EA group compared to the MCAO/R group. **b** Altered ALFF in the brain regions of MCAO/R + EA + antagomir-212-5p group compared to the MCAO/R + EA group. **c** Altered ALFF in the brain regions of MCAO/R + EA + antagomir-212-5p group compared to the MCAO/R + EA + antagomir-NC group. n = 8 per group. The voxel-level height threshold was *P* < 0.001(uncorrected) and the cluster-extent threshold were 10 voxels. M, MCAO/R group; M + EA, MCAO/R + EA group; M + EA + NC, MCAO/R + EA + antagomir-NC group; M + EA + antagomir, MCAO/R + EA + antagomir-212-5p group; L, left; R, right

Rs-fMRI was conducted on rats in the five groups to evaluate the effect of EA administration and antagomir-212-5p transfection on the brain. At 7 days postsurgery, the MCAO/R group displayed significantly lower ReHo values than the Sham group in the contralateral visual cortex and ipsilateral motor cortex. The MCAO/R + EA group showed significantly higher ReHo values than the MCAO/R group in areas of the ipsilateral posterior dorsal hippocampus, contralateral anterior dorsal hippocampus, contralateral subiculum hippocampus, contralateral dorsolateral thalamus, ipsilateral somatosensory cortex and ipsilateral corpus collosum. The MCAO/R + EA group showed significantly higher ReHo values than the MCAO/R + EA + antagomir-212-5p group in areas of the ipsilateral ventral hippocampus, contralateral anterodorsal hippocampus, ipsilateral amygdala and ipsilateral entorhinal cortex. The MCAO/R + EA + antagomir-NC group showed significantly higher ReHo values than the MCAO/R + EA + antagomir-212-5p group in areas of the ipsilateral orbitofrontal cortex, ipsilateral caudate putamen, ipsilateral dorsolateral thalamus, contralateral anterodorsal hippocampus and contralateral posterior dorsal hippocampus (Fig. 3; Table 1). In addition, when compared with the MCAO/R group, the MCAO/R + EA group displayed higher ALFF values in the ipsilateral caudate putamen and ipsilateral anterodorsal hippocampus. When compared with the MCAO/R + EA + antagomir-212-5p group, the MCAO/R + EA group displayed higher ALFF values in the ipsilateral ventral hippocampus and ipsilateral insular cortex. In addition, the MCAO/R + EA + antagomir-NC group displayed higher ALFF values in the ipsilateral somatosensory cortex compared with the MCAO/R + EA + antagomir-212-5p group (Fig. 4; Table 2).

**Table 1:** Difference in the regional homogeneity (ReHo) among groups 7 day postsurgery

Contrast				MNI Coordinates		
Name	Region Label	Extent	t-value	x	y	z
S > M	R_Cortex_Visual	185	6.580	49	26	5
	L_Cortex_Motor	10	4.831	-17	40	-49
M < M + EA	L_Hippocampus_Postero_Dorsal	15	-4.700	-50	5	7
	R_Hippocampus_Antero_Dorsal	30	-4.678	7	15	-25
	R_Hippocampus_Subiculum	14	-4.544	36	13	11
	R_Thalamus_Dorsolateral	11	-4.392	14	-1	-33
	L_Cortex_Somatosensory	18	-4.214	-65	-9	-51
	L_Corpus_Collosum	10	-4.141	-11	13	-53
M + EA > M + EA + antagomir	L_Hippocampus_Ventral	178	7.372	-32	-44	1
	R_Hippocampus_Antero_Dorsal	47	5.116	5	9	-25
	L_Amygdala	52	5.104	-38	-53	-21
	L_Cortex_Entorhinal	41	4.619	-67	-40	11
M + EA + NC> M + EA + antagomir	L_Cortex_Orbitofrontal	36	6.214	-5	-1	-95
	L_Caudate_Putamen	36	4.895	-36	-9	-65
	L_Thalamus_Dorsolateral	47	4.796	-13	-26	-11
	R_Hippocampus_Antero_Dorsal	10	4.632	7	7	-27
	R_Hippocampus_Postero_Dorsal	23	4.402	22	19	-7

Abbreviations: S, Sham group; M, MCAO/R group; M + EA, MCAO/R + EA group; M + EA + NC, MCAO/R + EA + antagomir-NC group; M + EA + antagomir, MCAO/R + EA + antagomir-212-5p group; L, left; R, right

**Table 2:** Difference in the amplitude of low-frequency fluctuations (ALFF) among groups 7 day postsurgery

Contrast				MNI Coordinates		
Name	Region Label	Extent	t-value	x	y	Z
M < M + EA	L_Caudate_Putamen	10	-5.097	-38	-7	-57
	L_Hippocampus_Antero_Dorsal	10	-4.957	-13	13	-25
M + EA > M + EA + antagomir	L_Hippocampus_Ventral	213	7.836	-30	-44	-1
	L_Cortex_Insular	16	6.655	-52	-11	-57
M + EA + NC> M + EA + antagomir	L_Cortex_Somatosensory	15	4.781	-17	28	-31

Abbreviations: MCAO/R group; M + EA, MCAO/R + EA group; M + EA + NC, MCAO/R + EA + antagomir-NC group; M + EA + antagomir, MCAO/R + EA + antagomir-212-5p group; L, left; R, right

### EA treatment decreases apoptosis via miR-212-5p

**Fig. 5:**
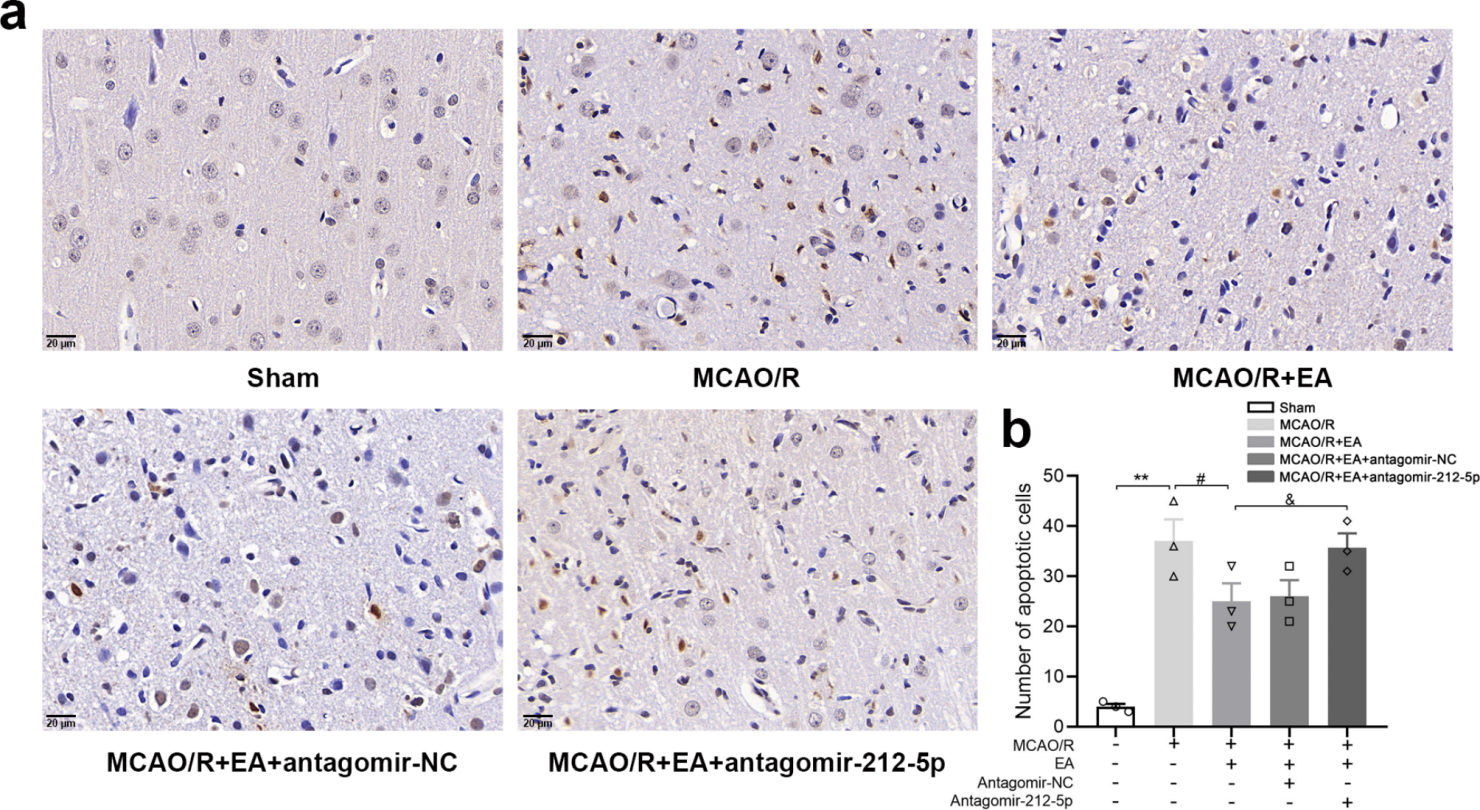
TUNEL detected cell apoptosis in the ischemic penumbra of rat brain tissue. **(a)** Representative images of TUNEL staining in the ischemic penumbra from different groups. **(b)** The number of TUNEL positive cells in the ischemic penumbra was calculated. Magnification ×400. Scale bar = 20 μm. The data are presented as the means ± SEM (n = 3 per group). ^*^P < 0.05 and ^**^P < 0.01 compared with the Sham group, ^#^*P* < 0.05 and ^##^*P* < 0.01 compared with the MCAO/R group, ^&^*P* < 0.05 and ^&&^*P* < 0.01 compared with the MCAO/R + EA group

Cell death was examined using TUNEL staining. As shown in Fig. 5, the data revealed a significant increase in the number of dead cells was observed in the ischemic penumbra 7 days after modeling compared with the Sham group (*P* < 0.001). After treatment with EA, cell death was significantly reduced compared with that in the MCAO/R group (*P* < 0.05). No significant differences were found between the MCAO/R + EA group and MCAO/R + EA + antagomir-NC group. Notably, a greater number of dead cells was observed in the rats treated with MCAO/R + EA + antagomir-212-5p than in those treated with EA alone (*P* < 0.05). The results demonstrated that EA treatment significantly reduced MCAO/R-induced cell death; however, antagomir-212-5p attenuated the effect of EA.

## Discussion

In recent years, many studies demonstrated that miRNAs contribute to the development of ischemic stroke, but whether they are participate in the regulatory process of EA in ischemic stroke remains to be explored. A rat model of cerebral ischemia reperfusion injury was used in this study to investigate the effect of EA and to explore the influence of antagomir-212-5p on this process. In our study, there was a deficits in gait, abnormal latency and amplitude and an increase in the number of dead cells in the MCAO/R group compared with the Sham group, and EA treatment partly reversed these phenomena. In addition, we found that focal cerebral ischemia–reperfusion induced several alterations in ALFF and ReHo metrics of the rat brain, and EA at the ST-36 and LI-11 acupoints promoted an increase in ALFF and ReHo values in areas of the brain related to motor or cognitive functions. However, transfection of antagomir-212-5p diminished the therapeutic effect of EA.

EA stimulation at LI11 combined with ST36 has an obvious effect on both neurogenesis and anti-apoptosis [22]. However, the protective mechanism of EA on ischemic stroke is far from understood. Several miRNAs have been reported to regulate neurogenesis and influence neuronal function [23]. Under normal conditions, miRNA expression exhibits extensive expression, tissue and cell specificity, temporal specificity and high conservation, and aberrant expression of miRNAs is involved in a variety of pathophysiological processes, indicating that miRNAs are important tools for the diagnosis and treatment of diseases [24]. MiR-212 is one of the members of the miR-132/212 family. The miR-132/212 is a well-known gene cluster located in neuronal cells and plays vital roles in the development, maturation, and function of neurons. Deregulation of this miRNA family has been linked to several neurological disorders, including Alzheimer’s and tauopathies [7, 25]. One study showed that in parkinsonian mouse models, miR-212-5p prevented the loss of dopaminergic neurons by targeting SIRT2 [26], another study indicated a decline in miR-212-5p levels after traumatic brain injuries, which are involved in neuronal cell death [27]. MiR-212 has been extensively described for its involvement in neuronal functions, however, the biology of its role in ischemic stroke progression remains unknown. A recent study by Chen et al. [28] reported that the downregulation of miR-212 in the MCAO model. MiR-212 suppressed matrix metallopeptidase 9 to block the Notch signaling pathway, thus promoting the regeneration of vascular tissue and endothelial cell function after ischemic stroke. Previous studies have shown that miR-212-5p might serve as a potential biomarker and therapeutic target for ischemic stroke. Overexpression of miR-212-5p leads to the downregulation of Caspase 7 (CASP7) and prevents neuronal death [29]. Whether miR-212-5p is involved in the EA-mediated therapeutic effect in ischemia–reperfusion rats requires further exploration. In this study, gait parameters determined by Catwalk gait analysis were improved after EA treatment, and electrophysiology demonstrated that EA ameliorated the abnormal latency and peak amplitude. In addition, EA treatments immediately after MCAO/R were anticipated to decrease neuronal death. However, the application of antagomir-212-5p hindered the treatment efficacy of EA. Thus, the effect of EA treatment might have partly occurred through miR-212-5p.

With the rapid development of fMRI, it can be used to investigate the therapeutic mechanism of acupuncture in terms of brain science. Schaechter et al. reported that acupuncture was performed in ischemic stroke patients with hemiparesis for 10 weeks, and fMRI image were obtained before and after treatment. Results showed that acupuncture treatment could improve the motor function of hemiplegic limbs after ischemic stroke by increasing the activity of the affected motor cortex [30]. ALFF has been suggested to be associated with local neuronal activity in the resting state and reflects regional energy metabolism and efficient signal transfer at chemical synapses [31]. The ALFF index was found to be reliable and sensitive for describing various brain diseases, such as stroke, Parkinson’s disease, and posttraumatic stress disorder [32]. An fMRI study revealed that acupuncture can specifically enhance the functional activities of language processing, sensory integration and motor coordination-related brain regions in the dominant hemisphere of patients [33]. Another fMRI study found decreased ALFF values in the auditory cortex, cingulate gyrus, dorsal thalamic nucleus, hippocampus, motor cortex, preimmune cortex, retrosplenic cortex, and sensory cortex of rats subjected to MCAO compared with the Sham group. However, EA at DU20 and DU24 acupoints improves cognitive dysfunction, and activation of brain regions may be responsible for its protective effects, such as the hippocampus, posterior splenic cortex, cingulate gyrus, preimmune cortex and sensory cortex [34]. According to the present study, ALFF was increased in the ipsilateral caudate putamen and ipsilateral anterodorsal hippocampus after EA intervention compared with the model group. Significant ALFF attenuation in the ipsilateral ventral hippocampus, ipsilateral insular cortex and somatosensory cortex was discovered in the MCAO/R + EA + antagomir-212-5p group compared with rats treated with EA. The putamen and caudate belong to the striatum, which is a subcortical structure that has a critical role in cognition and motor control, associative behaviors and behavioral flexibility [35]. ALFF values for the somatosensory cortex and hippocampus were also affected by EA treatment. The hippocampus plays an important role in regulating emotionality and cognitive processes, including learning, consolidation, and retrieval of information [36]. Indeed, it has been shown that reconstruction of cognitive-related brain regions after cerebral infarction is important for the recovery of motor function [37].

ReHo reflects the local synchrony of neural activity, an increase in ReHo indicates the uniformity of neuronal activity in regional areas of the brain, and abnormalities in ReHo may reflect disorders of local brain functioning. In a study enrolling a total of 21 ischemic stroke patients and 21 healthy controls who received rs-fMRI, ReHo analysis performed for each subject revealed that ReHo values were decreased in several brain regions such as cerebellumthe right dorsolateral prefrontal cortex in patients with ischemic stroke compared with healthy subjects [38]. A previous study has shown that compared with healthy subjects, ReHo values in the bilateral anterior cingulate cortex and left posterior cingulate cortex/precuneus are decreased in patients with cognitive dysfunction after stroke [39]. The present findings are in agreement with previous studies. The MCAO/R group exhibited significantly decreased ReHo values in the ipsilateral motor cortex compared with the Sham group. Ischemia–reperfusion-induced brain injury attenuated the activity of a large proportion of neurons in motor brain regions, reducing synchrony in the spontaneous activity of neurons and thus impairing the preparation, execution, coordination and stabilization of motor function.

In addition, we identified that treatment with EA significantly increased ReHo values within the ipsilateral posterior dorsal hippocampus, contralateral anterodorsal hippocampus, contralateral subiculum hippocampus, contralateral dorsolateral thalamus, ipsilateral somatosensory cortex and ipsilateral corpus collosum compared with those of MCAO/R rats. The main function of the thalamus is to integrate and process motor and sensory information [40]. The somatosensory cortex receives sensory inputs, and the motor cortex controls the movement of muscles, which are key areas in the cortical sensorimotor network [41]. The corpus callosum plays a key role in the transfer and integration of sensory, motor and cognitive information between the cortices [42]. We also found that EA increased neuronal activity in the bilateral hippocampus. Therefore, EA at ST36 and LI11 could also facilitate cognitive function recovery. However, the bilateral hippocampus, ipsilateral amygdala, entorhinal cortex, orbitofrontal cortex and caudate putamen showed decreased ReHo in the antagomir-212-5p group compared with the rats treated with EA. The amygdaloid is part of the limbic system and is involved in memory and emotional behavior [43]. The entorhinal cortex is widely regarded as the hub of cortico-hippocampal circuits and has also been found to be involved in cognition [44]. The orbitofrontal cortex (OFC) is a prefrontal cortex region that has been implicated in multisensory integration [45]. Taken together, we speculated that EA might play a protective role in motor and cognitive function in MCAO/R rats by reactivating motor and cognition-related brain regions. However, the protective effect of EA was attenuated by pretreatment with antagomir-212-5p.

The present study has several limitations. First, further studies with an expanded sample size are needed to provide additional experimental evidence for EA treatment of ischemic stroke. Second, considering the mortality of the rats, contiguity neuroimaging scanning was not performed after surgery. Third, the observation was performed for merely 7 days in the subacute stage of ischemic stroke, and no long-term follow-ups were conducted. Despite the aforementioned limitations, the current study, with the administration of EA and antagomir-212-5p, implicated several cerebral regions in the pathogenesis of ischemic stroke.

## Conclusion

In conclusion, the brain following cerebral ischemia reperfusion was accompanied by impaired neural activities in brain regions related to cognitive and motor function, a reduced conduction velocity, severe neuronal loss, and functional changes associated with motor function decline. Acupuncture at the LI11 and ST36 acupoints could promote the activation of neuronal function in cognitive and motor-related brain areas, improve conduction velocity, and decrease neuronal death in the ischemic penumbra, thereby promoting the recovery of motor dysfunction. The miR-212-5p inhibitor attenuated the protective effects of EA treatment, which implied that EA treatment could partly reverse this phenomenon through miR-212-5p. This study provides a basis for exploring the mechanism of action of EA treatment and identifying a target for EA treatment.

## Data Availability

The datasets used and/or analyzed during the current study are available from the corresponding author on reasonable request.
